# Epidemiology of cardiac amyloidosis in Germany: a retrospective analysis from 2009 to 2018

**DOI:** 10.1007/s00392-022-02114-y

**Published:** 2022-10-14

**Authors:** Svenja Ney, Peter Ihle, Thomas Ruhnke, Christian Günster, Guido Michels, Katharina Seuthe, Martin Hellmich, Roman Pfister

**Affiliations:** 1 Faculty of Medicine, Department III of Internal Medicine, University of Cologne, University Hospital Cologne, Kerpener Str. 62, 50937 Cologne, Germany; 2 Medical Faculty, PMV Forschungsgruppe, University of Cologne, Cologne, Germany; 3 AOK Research Institute, WIdO, Berlin, Germany; 4 Klinik für Akut- und Notfallmedizin, St.-Antonius-Hospital, Eschweiler, Germany; 5 Institute for Medical Statistics and Bioinformatics, University of Cologne, Cologne, Germany

**Keywords:** Cardiac amyloidosis, Light-chain amyloidosis, Transthyretin amyloidosis, Amyloid cardiomyopathy

## Abstract

**Background:**

Improved imaging modalities contributed to increasing awareness of cardiac amyloidosis. Contemporary data on frequency trends in Germany are lacking.

**Methods:**

In a retrospective study using health claims data of a German statutory health insurance, patients with diagnostic codes of amyloidosis and concomitant heart failure between 2009 and 2018 were identified.

**Results:**

Prevalence increased from 15.5 to 47.6 per 100,000 person-years, and incidence increased from 4.8 to 11.6 per 100,000 person-years, with a continuous steepening in the slope of incidence trend. In patients with amyloidosis and heart failure age and proportion of men significantly increased, whereas the frequency of myeloma and nephrotic syndrome significantly decreased over time. Median (IQR) survival time after first diagnosis was 2.5 years (0.5–6 years), with a 9% (95% CI 2–15%, *p* = 0.008) reduced risk of death in the second compared to the first 5 years of observation. In the 2 years prior and 1 year after diagnosis, mean total health care costs were 6568 €, 11,872 € and 21,955 € per person and year.

**Conclusion:**

The rise in cardiac amyloidosis has continuously accelerated in the last decade. Considering the adverse outcome and high health care burden, further effort should be put on early detection of the disease to implement available treatment.

**Supplementary Information:**

The online version contains supplementary material available at 10.1007/s00392-022-02114-y.

## Introduction

Historically cardiac amyloidosis (CA) was regarded an orphan disease with detrimental outcome and low awareness within the medical community [1]. Patients with CA were gathered at few specified centers which provided the existing evidence on frequency and clinical course of the disease [2–4]. In the last two decades, advances in cardiac imaging techniques substantially contributed to an increased recognition of CA in routine practice [5, 6]. Non-invasive diagnostics using multimodality imaging lowered the threshold to seek diagnosis of CA in elderly patients [7]. Findings from screening and autopsy studies showed a high rate of undetected CA in elderly patients with heart failure and preserved ejection fraction. This highlights the systematic underreporting of CA prevalence in earlier studies [8, 9] and might have increased readiness of physicians to initiate diagnostics in those patients.

The most important milestone revising the perception of CA was the approval of disease modifying drugs for the two dominating subtypes—immunoglobulin light chain (AL) and transthyretin amyloidosis (ATTR) [10, 11]. Additionally, highly effective drugs for ATTR are at advanced stages of clinical evaluation (HELIOS-B [NCT04153149], CARIO-TTRansform [NCT04136171] and [12]).

Taken together, there is increasing attention of CA with respect to epidemiology and impact on the health care system particularly in the context of available but costly treatment options. Existing data are either outdated [13] or derived from highly selected patient populations [3, 4, 14]. Here, we describe epidemiology during the last decade and impact on health care system of CA using a population-based approach with health insurance claims data of the largest German statutory health insurance.

## Methods

### Data set and sample selection

We performed a retrospective cohort study with health insurance claims data of the AOK (Allgemeine Ortskrankenkasse) provided by the AOK Research Institute. AOK provides statutory health insurance for roughly 32 percent of the German population (26.5 Mio members in 2018). Membership is open to anyone regardless of factors such as professional affiliation, income, age or comorbidities [15]. The study complies with the *Declaration of Helsinki*. Ethical approval was not necessary since patient data were completely anonymized.

The initial data set included all individuals insured with an International Classification of Diseases-10th revision (ICD-10) diagnosis code of amyloidosis [E85 inpatient primary or secondary diagnosis, or outpatient diagnosis in at least two quarters of one year], from January 1, 2008 to December 31, 2019. Survival information was available until the end of 2019. For our analysis, we selected a subsample with presumable cardiac involvement defined by at least one heart failure diagnosis [I11.0, I13.0, I13.2, I42.1, I42.2, I42.5, I42.8, I43.1, I50] within four quarters before, three quarters after or in the quarter of first diagnosis of amyloidosis. Furthermore, selected patients needed to be at least 60 years old at the date of the first documented diagnosis of amyloidosis to more specifically focus on wild-type ATTR cases which have the highest expected underreporting and the highest rate of cardiac involvement.

A cohort for calculation of incidence was defined as patients with first diagnosis of CA, i.e., no diagnosis of amyloidosis for at least the previous four quarters. We therefore excluded patients with a baseline period (pre-observational) of less than 1 year, i.e., with a first diagnosis of amyloidosis before January 1, 2009. The quarter of first diagnosis of amyloidosis was referred as baseline. We also excluded patients with an outcome period (post-observational) of less than 1 year, i.e., with a first diagnosis of amyloidosis after December 31, 2018, to allow validation of amyloidosis and heart failure diagnosis.

Patients with CA were counted for the cohort for calculation of prevalence in each year they were alive and insured for at least 1 day, starting with the year of first diagnosis of CA. This approach was chosen because CA is a chronic persistent disease that cannot be cured except by transplantation. Defining patients with CA only if a diagnostic code of CA is present in that year will result in underestimation of prevalence since diagnoses in claims data are often coded in the context of diagnostic or therapeutic procedures and can be lost subsequently.

For analysis of health care costs, hospital admissions and outpatient introduction to physicians only patients with first diagnosis of CA in 2018, and continuous insurance 2 years before and 1 year after first diagnosis of CA were considered to provide most contemporary and consistent results.

### Study covariates

Clinical baseline characteristics were assessed by ICD-10 codes from the year before first diagnosis of amyloidosis. Health care expenditures (for inpatient treatment, outpatient treatment, drugs, medical adjuvants, and cumulative costs), number of hospital admissions and number of outpatient physician contacts were calculated by year. Number of outpatient physician contacts was approximated by the sum of numbers of days with an accounting for medical services per physician. The quarter with the first diagnosis of CA was not considered for health care impact analysis.

### Statistics

Prevalence and incidence rate by 100.000 person-years were calculated for each year by dividing the number of patients with prevalent and incident CA per the total insurance time of persons ≥ 60 years within the respective year. Rates presented for the total cohort were standardized on the basis of estimated age- and gender-specific prevalence rates according to the age and gender distribution of the German population of each year (derived from the Federal Office of Statistics, www.destatis.de) and, in supplementary analysis, of the starting year. Changes in the slope of increase of incidence over time were examined using an ordinary least-squares regression-based approach for interrupted time-series analysis. Baseline characteristics were presented as percentage for categorical variables and median (interquartile range IQR) or mean (standard deviation SD) for continuous variables as appropriate. Characteristics of patients with first diagnosis of CA were compared across years using a non-parametric test according to Cuzick which is an extension from the Wilcoxon rank-sum test and regression analysis. Mortality was described with the use of Kaplan–Meier estimates, and patient groups by period of first diagnosis of CA were compared using Cox proportional hazard analysis adjusted for age and gender. Follow-up time was calculated from first diagnosis of CA till death, end of observation (31st December 2019) or end of insurance status, whichever came first, and was available only on a quarter-base due to protection of data privacy. For patients deceasing or withdrawing insurance status within the baseline quarter, a fixed observational time of 0.5 quarter was assigned. Statistical analyses were performed with the use of STATA/SE version 12.1 (Tx, USA).

## Results

### Epidemiology 2009–2018

**Fig. 1:**
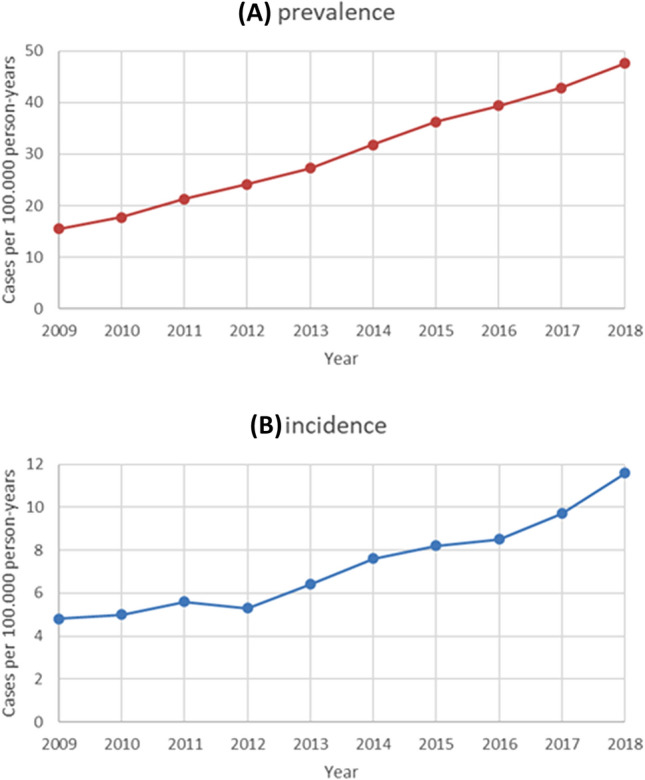
Prevalence (**A**) and incidence (**B**) trends of patients with amyloidosis with concomitant heart failure, 2009–2018, standardized to the age and gender distribution of the German population

There were 5618 patients with first diagnosis of CA and 8279 patients with any diagnosis of CA in the 10-years observation period. Of the patients with first diagnosis of CA, 7% had a code for hereditary types of amyloidosis and 89% had a code for organ limited or unspecified amyloidosis (Supplementary Table 1). Prevalence continuously increased from 15.5 (95% CI 14.7–16.4) in 2009 to 47.6 (95% CI 46.1–49.1) per 100,000 person-years in 2018, and incidence increased from 4.8 (95% CI 4.3–5.3) to 11.6 (95% CI 10.9–12.4) per 100,000 person-years (Fig. 1). The slope of the incidence curve increased in years 2012–2013 and 2016–2017, with an estimated yearly increase in incidence of 0.4 per 100.000 person-years from 2009 to 2012, 1.0 per 100.000 person-years from 2012 to 2016 and 1.6 per 100.000 person-years from 2016 to 2018, respectively. Standardization of rates to the gender- and age distribution of the starting year (2009) to account for demographic changes of the German population over time did not virtually change the observed trend (Supplementary Fig. 1).

**Fig. 2:**
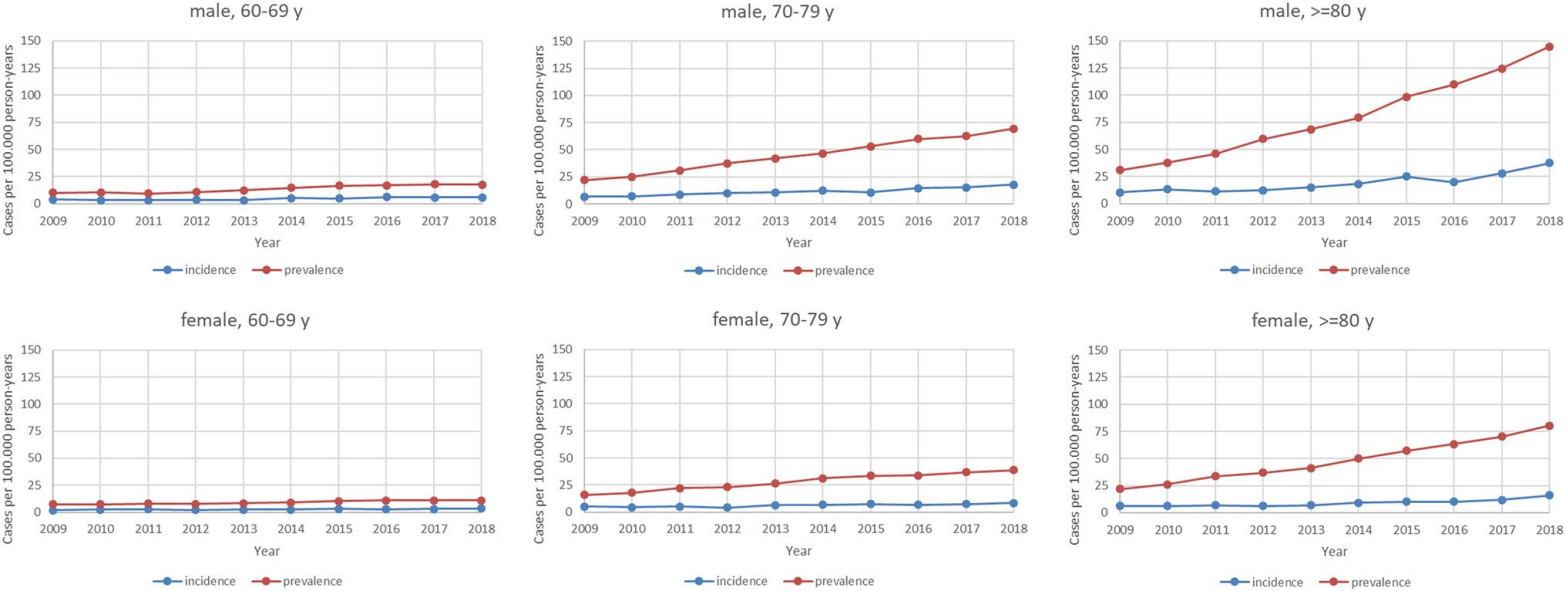
Variation in the annual number of patients with amyloidosis and concomitant heart failure by age, gender, 2009–2018

Variations in the annual numbers of prevalence and incidence by age categories and sex are shown in Fig. 2. Both the absolute prevalence and incidence as well as the relative increase of prevalence and incidence were more pronounced in men and very elderly people. Men of 80 years or older showed a prevalence of 144.9 (95% CI 136.1–154.2) and an incidence of 37.5 (95% CI 33.2–42.4) per 100.000 person-years in 2018, with an increase in prevalence and incidence in 2018 compared to 2009 by factor 4.7 and 3.7, respectively. In contrast, women of 60–69 years of age showed a prevalence of 10.9 (95% CI 9.4–12.7) and an incidence of 3.3 (95% CI 2.5–4.3) per 100.000 person-years in 2018, with an increase compared to 2009 by factor 1.5 and 1.8, respectively.

At the time of first diagnosis of amyloidosis patients had a mean (SD) age of 77 (8) years, with a significant increase from 76 (7) years in 2009 to 79 (8) years in 2018 (Table 1). 54% of patients were male, with an increase from 48% in 2009 to 58% in 2018. The majority of patients had a hospitalization for heart failure within the previous year and had a hospitalization in the quarter of the first diagnosis of amyloidosis. In terms of comorbidity there was a substantial increase in atrial fibrillation from 27% in 2009 to 41% in 2018, and in renal dysfunction from 38% in 2009 to 45% in 2018. Amyloidosis associated comorbidity showed a significant increase in polyneuropathy from 16% in 2009 to 23% in 2018, and a significant decrease in myeloma and nephrotic syndrome from 8% in 2009 to 4% in 2018 and 4% in 2009 to 1% in 2018, respectively.

**Table 1:** Baseline characteristics of patients newly diagnosed with cardiac amyloidosis

	2009	2018	P for trend	Total
*N*	375	901		5618
Age, mean (SD)	76 (7)	79 (8)	< 0.001	77 (8)
Age categories, *n* (%)				
60–69 years	75 (20)	133 (15)		968 (17)
70–79 years	171 (46)	294 (33)		2316 (41)
80 years or above	129 (34)	474 (53)		2334 (42)
Gender, male *n* (%)	179 (48)	520 (58)	< 0.001	3058 (54)
Prior heart failure hospitalization < 12 months	251 (67)	587 (65)	0.62	3675 (65)
NYHA class available	87	371		1929
I/II	16 (18)	128 (34)	0.001	586 (30)
III	38 (44)	141 (38)		766 (40)
IV	33 (38)	102 (27)		577 (30)
Inpatient diagnosis of amyloidosis	290 (77)	739 (82)	< 0.0001	4420 (79)
Past medical history, *n* (%)				
Myocardial infarction	32 (9)	130 (14)	0.003	699 (12)
Coronary heart disease	183 (49)	452 (50)	0.26	2743 (49)
Hypertension	316 (84)	826 (92)	< 0.0001	5061 (90)
Diabetes mellitus	139 (37)	356 (40)	0.75	2260 (40)
Atrial fibrillation	103 (27)	367 (41)	< 0.0001	1975 (35)
Stroke	33 (9)	111 (12)	0.07	681 (12)
Renal dysfunction	144 (38)	405 (45)	< 0.0001	2330 (41)
Chronic obstructive pulmonary disease	82 (22)	198 (22)	0.14	1199 (21)
Dementia	41 (11)	159 (18)	< 0.0001	841 (15)
Anemia	74 (20)	190 (21)	0.71	1101 (20)
Polyneuropathy	60 (16)	204 (23)	< 0.0001	1009 (18)
Carpal tunnel syndrome	17 (5)	72 (8)	0.003	378 (7)
Orthostatic hypotension	14 (4)	54 (6)	0.04	294 (5)
Multiple myeloma	30 (8)	33 (4)	< 0.0001	274 (5)
Monoclonal gammopathy of unknown significance (MGUS)	23 (6)	42 (5)	0.90	259 (5)
Myelomatosis or MGUS	44 (12)	62 (7)	0.01	457 (8)
Nephrotic syndrome	14 (4)	13 (1)	< 0.0001	133 (2)

Median (IQR) survival time after first documented diagnosis of amyloidosis was 2.5 years (0.5–6 years). Cumulative 1-year and 2-year survival was 70.0% (95% CI 68.7–71.1) and 56.9% (95% CI 55.6–58.2). Survival significantly improved in the second half of the observation period (2014 till 2018) compared to the first half (2009 till 2013) when adjusting for age and gender (hazard ratio 0.91, 95% CI 0.85–0.98, *p* = 0.008). Patients diagnosed in 2018 had the highest one-year survival (72.9%, 95% CI 0.70–0.76).

### Health care impact in patients of 2018

**Fig. 3:**
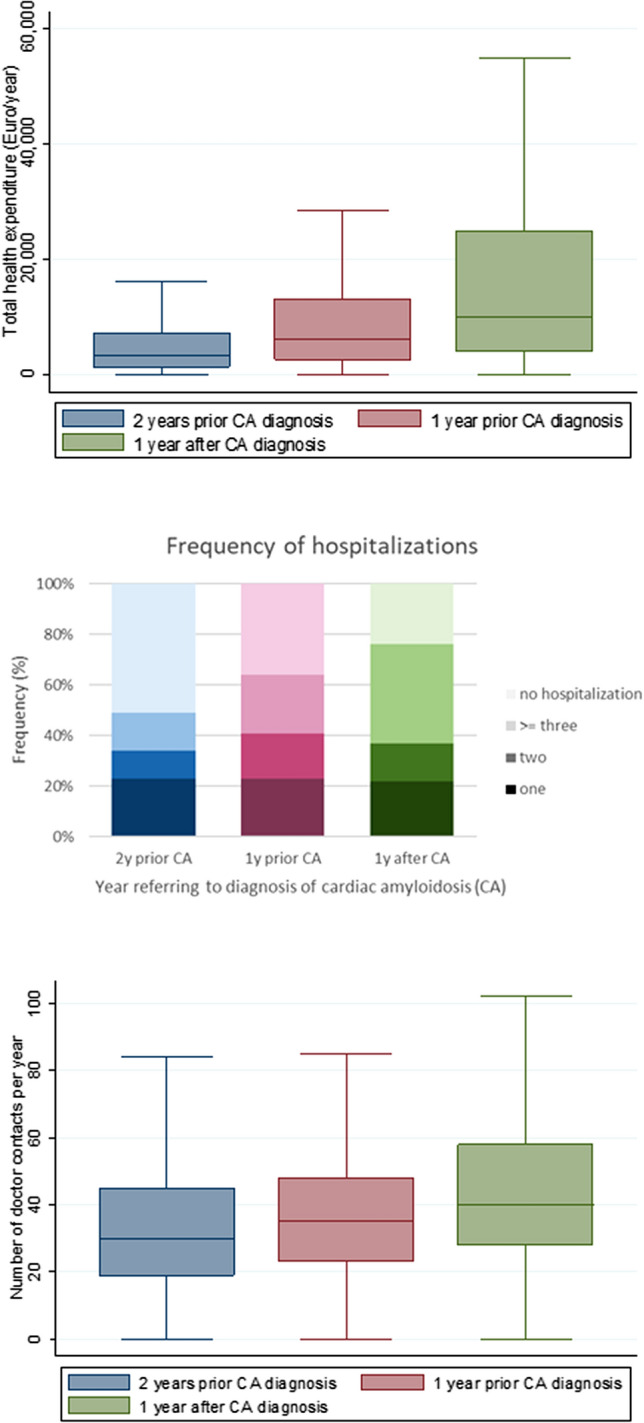
Total health expenditure (**A**), number of hospitalizations (**B**) and number of contacts to outpatient doctors (**C**) for patients diagnosed with amyloidosis and concomitant heart failure in 2018

Figure 3A shows total health care costs in patients with diagnosis of CA in 2018 for 2 years before and in the year after the diagnosis. Median (IQR) total health care costs per year increased during disease progression from 3280 € (1365–7229, mean 6568 €) 2 years prior to diagnosis, 6013 € (2626 to 13,085, mean 11,872 €) 1 year prior to diagnosis and to 9873 € (3922–24,714, mean 21,955 €) in the year after diagnosis. Main contributor of medical costs were expenditures for hospitalizations ranging from 44.6 to 68.9% of total costs.

Two years prior to the first diagnosis of CA about half of patients had at least one hospitalization per year (Fig. 3B). One year prior and in the year after the diagnosis of CA 23% and 39% of patients had three or more hospitalizations per year. The number of outpatient doctor contacts per year increased from a median (IQR) of 30 (19–45) 2 years prior to the diagnosis to 40 (28–58) in the year after the first diagnosis (Fig. 3C).

## Discussion

We report epidemiology throughout the last decade and current health care impact of patients with diagnosis of amyloidosis and concomitant heart failure in a large elderly German population. Prevalence and incidence continuously increased from 15.5 and 4.8 per 100.000 person-years in 2009 to 47.6 and 11.6 in the last year of observation 2018. Temporal changes of patient characteristics with respect to gender, age and comorbidities suggest wild-type transthyretin amyloidosis as the main driver of increasing incidence. All-cause mortality was high with only mild improvement in the last 10 years and yet a 1-year mortality of 27% in patients diagnosed most recently in 2018. Health care burden in these patients is high with mean total expenditures of more than 20,000 € in the first year after diagnosis.

Gilstrap et al. were the first to provide population-based frequency data on CA using Medicare data from the Unites States. Between 2000 and 2012 prevalence and incidence of CA continuously increased with a particular inflection point between years 2006 and 2007. We extend these findings in showing that the increase in CA prevalence and incidence further persists till 2018 with an almost triplication within one decade. Recently, a nationwide study from Denmark also reported an overall increase in CA throughout the last two decades [16]. However, our reasonably large population size which comprises five-times more people than the Danish population allowed a detailed analysis on a yearly basis. The temporal trend of CA incidence suggested an accelerating increase with additional inflection points at years 2012/2013 and 2016/2017. The increasing number of CA is unlikely to reflect a true increase in the number of affected patients but is rather the consequence of increased disease awareness and identification of patients through simplified diagnostics. In support of this, the use of bone scintigraphy to diagnose CA remarkably started to rise in 2011 [17]. Notably, our frequency trend indicates that the massive under-detection of CA has not yet been solved. Screening studies showed a prevalence of ATTR within elderly patients with heart failure and preserved ejection fraction between 5 and 13% [8, 18, 19]. Importantly, the rate of patients with mild heart failure reflected by NYHA class I or II increased over time suggesting that not only more patients are identified but patients are also identified at an earlier disease stage and might benefit from tafamidis therapy.

The data source used here and in other population-based studies does not allow a reliable classification of underlying subtypes of amyloidosis on a person level since specific diagnostic codes for distinct molecular amyloidosis subtypes are lacking. However, cardiac involvement in amyloidosis is almost entirely restricted to AL and ATTR with rare cases of amyloid A (AA) amyloidosis. Patients with AL are usually 10 years younger than wild-type (wt) ATTR patients, without a clear predominance in gender and the majority has renal involvement [20]. Patients with AA amyloidosis have a median age of 65 years, predominant renal involvement and a male–female ratio of 1:3 [21]. Patients with genetic ATTR usually are also younger than patients with wtATTR with a mean age at diagnosis of 66 years [22]. Furthermore, genetic ATTR shows cardiac involvement in only about a quarter of patients. Taken together, when considering the clinical and demographic phenotype of the distinct amyloidosis subtypes, the recent increase of patients with amyloidosis and concomitant heart failure clearly points to underlying wtATTR. This is in line with the observation of a large UK referral center where wtATTR showed an exponential increase in the last decade [3].

Our findings highlight the still detrimental impact of CA on health outcome by showing a median survival of 2.5 years in our unselected real-world population which did not change substantially over the last decade. The minor survival improvement might be multifactorial with diagnosis of CA at earlier stages and shifts in underlying amyloidosis subtypes as potential explanations. Important to note, potential beneficial effects of new drugs such as tafamidis for ATTR cardiomyopathy and daratumumab for AL amyloidosis cannot yet be visible in our analysis since approval in Europe was as recently as in 2020 and 2016. Albeit survival rates in AL-CA derived from referral centers are comparably low as in our study [23, 24], prognosis of ATTR-CA reported by referral centers [3, 14, 25] or in randomized controlled trials [11] are markedly higher. Presuming that the majority of patients in our cohort have underlying wtATTR, the discrepancy in outcome with earlier studies are quite remarkable and underscores the value of population-based analysis approaches.

Patients with amyloidosis and concomitant heart failure showed frequent contacts with the health care system, particularly within the outpatient setting but also via hospital admissions. Median number of outpatient doctor contacts was 40 per year in the most recent patients with first diagnosis of CA in 2018. This is markedly higher than the average in Germany which is 19 per year in the age group of 60–64 years and 30 per year in the age group of 80–84 years [26]. This underlines the high medical burden of these elderly patients with cardiac amyloidosis. On the other hand, the regular contact with physicians already several years prior to the diagnosis of amyloidosis provides the chance to prepone the diagnosis and in consequence treatment initiation. Nonetheless, this requires further increase in disease awareness and structured algorithms for early detection including validated red flags. The major economic burden of cardiac amyloidosis is derived from hospitalizations which comprise more than two thirds of the total health expenditures. Albeit health behaviors, organization of health care systems and costs differ across countries, the magnitude of total spending per person for example in the context of complex elderly heart failure patients is comparable across central and northern European countries [27]. Hence, our cost estimates might be used for future cost-effectiveness analysis in the context of new drug treatments.

A major strength of our study is that claims data are less prone to selection bias compared to primary data from registers and specialized hospitals as already discussed. Additionally, CA yet is a rare disease and data sources like ours are needed to achieve a sufficiently large population for analysis.

### Limitations

Important limitations of the study need to be considered. Results must be interpreted in the context of a dominantly white population. Albeit data on race are not available in our dataset, according to the total German population, the portion of blacks is neglectable. These and other demographic characteristics may partly explain differences in absolute prevalence and incidence values between ours, the U.S. and the Danish study. There are few differences with respect to demographics and comorbidity between the examined cohort derived from a single insurance company and the total German population [29]. Nonetheless, most important for accurate estimates of prevalence and incidence in strata of age and gender are sufficiently large numbers in these group which is guaranteed given that our cohort comprised almost one third of the German population. The use of health insurance data comes along with several limitations with respect to restricted availability of laboratory and biometric data, and the variability in the management of coding across institutions all of which attenuates the quality of data and might be of particular impact in the setting of rare diseases. For instance, the combination of diagnostic codes for amyloidosis and heart failure is only an approximation to define CA. Since heart failure symptoms usually predate the diagnosis of amyloidosis in patients with CA, the latter approach will rather cause overestimation of CA frequency by falsely counting patients with preexisting heart failure due to other reasons than amyloidosis. Nonetheless, the trend in total heart failure prevalence in Germany over the last decade is rather flat and cannot explain the increases observed for CA [28]. On the other hand, rare diseases such as amyloidosis in general might be rather under-coded which leads to underestimation of CA prevalence and incidence as reported for other rare disease [30]. Nonetheless, assuming that these general limitations of the data source do not change relevantly within 10 years our findings on the temporal trend will not be impacted relevantly. Finally, our approach to define CA patients results in an underestimation in the first two to three years of the observation period since patients with diagnosis of CA prior to 2008 who lost their diagnostic code during this time cannot be detected. In consequence, the absolute increase in prevalence between 2009 and 2018 will be slightly smaller than reported but the slope of increase during the last 8 years remains the same.

In conclusion, the temporal trend in the last decade shows a continuously accelerating increase in prevalence and incidence of amyloidosis with concomitant heart failure in a large part of the German population. Inferring from the patient characteristics increased detection of patients with wtATTR might be the main driver of this observation. In the light of available effective treatments and considering the major impact of CA on patient and health care systems further effort is warranted to intensify early detection of patients with CA.

## Supplementary Information

Below is the link to the electronic supplementary material.

Supplementary Figure 1: Prevalence (A) and incidence (B) trends of patients with amyloidosis with concomitant heart failure, 2009–2018, standardized to the age and gender distribution of the German population (red and blue) and additionally to the starting year 2009 (green) (JPG 58 KB)

Supplementary file2 (DOCX 12 KB)
