# Supplementary figures and images for: Epidemiology of cardiac amyloidosis in Germany: a retrospective analysis from 2009 to 2018
Source: Clin Res Cardiol. 2022 Oct 14;112(3):401–8. doi: 10.1007/s00392-022-02114-y (PMC9998316; doi:10.1007/s00392-022-02114-y)

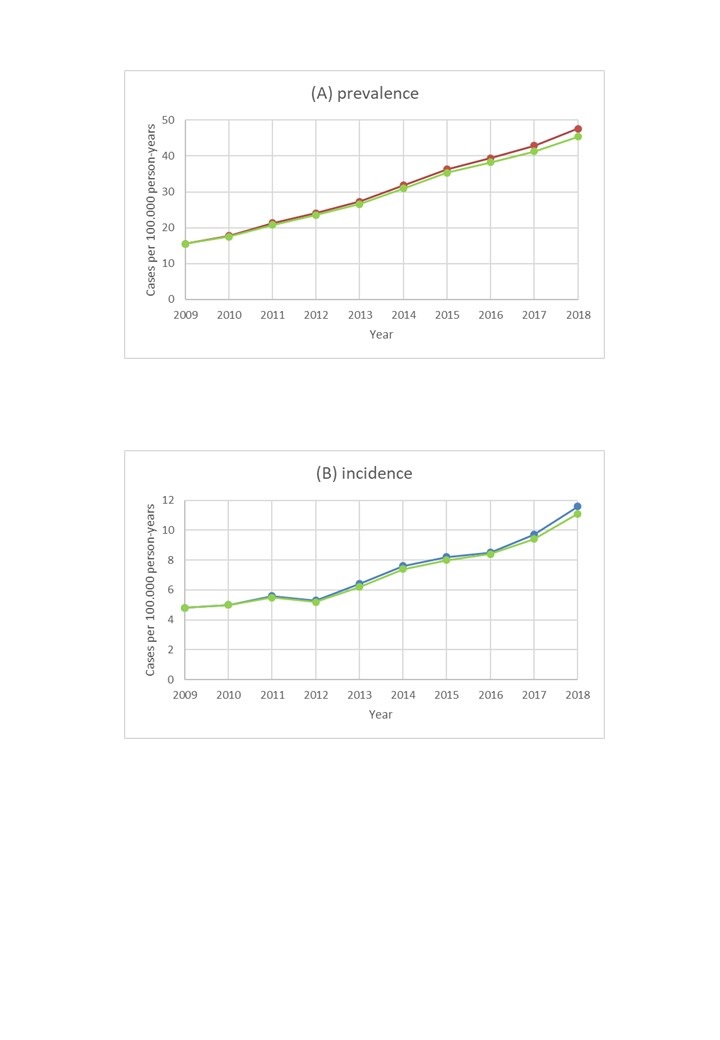

Supplement: Supplementary file 1 — Supplementary Figure 1: Prevalence (A) and incidence (B) trends of patients with amyloidosis with concomitant heart failure, 2009–2018, standardized to the age and gender distribution of the German population (red and blue) and additionally to the starting year 2009 (green) (JPG 58 KB) [file 392_2022_2114_MOESM1_ESM.jpg]
