# Supplementary material for: Epidemiology of cardiac amyloidosis in Germany: a retrospective analysis from 2009 to 2018
Source: Clin Res Cardiol. 2022 Oct 14;112(3):401–8. doi: 10.1007/s00392-022-02114-y (PMC9998316; doi:10.1007/s00392-022-02114-y)
Supplement: Supplementary file 2 — Supplementary file2 (DOCX 12 KB) [file 392_2022_2114_MOESM2_ESM.docx]

Supplementary Table 1: Frequency of different codes reflecting diagnosis of amyloidosis with patients with first diagnosis of cardiac amyloidosis.

| Code | Diagnosis | Frequency* |
| --- | --- | --- |
| E 85.0 | Non-neuropathic heredo-familial amyloidosis | 307 (5.5%) |
| E 85.1 | Neuropathic heredo-familial amyloidosis | 83 (1.5%) |
| E 85.2 | Heredo-familial amyloidosis, not specified | 30 (0.5%) |
| E 85.3 | Secondary systemic amyloidosis | 526 (9.4%) |
| E 85.4 | Organ limited amyloidosis | 3,152 (56.1%) |
| E 85.8 | Other amyloidosis | 892 (15.9%) |
| E 85.9 | Amyloidosis, not specified | 1,848 (32.9%) |

*adds up to more than 100% since some patients has more than one code.
